# A Method of Planning Disaster Emergency Rescue Paths in Road-Free Environment

**DOI:** 10.1155/2022/2987852

**Published:** 2022-01-31

**Authors:** Qing Xu, Shisheng Feng, Qun Sun, Xinming Zhu, Ruoxu Chen, Xiaoyang Lihua, Beibei Wu

**Affiliations:** School of Geospatial Information, University of Information Engineering, Zhengzhou, Henan 450001, China

## Abstract

The occurrence of natural disasters has caused great loss of lives and properties that affected many people. To improve the efficiency of the emergency rescue efforts, the problem of road planning for emergency workers, vehicles, and other mobile objects should be overcome. Due to the characteristics of the emergency planning for rescue paths, timely, effective, and rapid efforts should be taken into effect based on geographical data in the road-free environment. This paper analyzes trafficability, researches both access ability and traffic efficiency, and uses traffic efficiency concerning the road-free environment as an influential weight and timeliness to improve the A^∗^ algorithm, which realizes the rapid planning of the mobile objects' emergency rescue paths in the road-free environment, providing decision-making services for the rescue path planning of disaster emergencies in the environment of no road network. Finally, the experiments were carried out using available data for Shuozhou City, Shanxi Province. The results show that the research can provide a reference for the rescue path planning of disaster emergencies and has contributed to theoretical and applicable aspects of the research.

## 1. Introduction

Typhoons, earthquakes, floods, and other natural disasters could occur, causing heavy losses to the lives and properties of many people [1]. Available technical means have not yet been able to effectively prevent and hinder the occurrence of these natural disasters; only by taking high-efficient disposal decisions and emergency response can efforts effectively mitigate losses caused by natural disasters [2]. To improve the efficiency of the emergency rescue and ensure the safety of the people affected by the disaster and distribute the supplies, it is first necessary to solve the path planning problem of disaster relief personnel, vehicles, and other mobile objects [3].

Planning researches of conventional emergency rescue paths [4–7] are mostly based on the identified road network, rely mainly on experience, and conduct qualitative or semiquantitative analysis based on time, cost, risk, and other aspects, which do not emphasize the emergency rescue characteristics such as the weak economy and strong timeliness. Moreover, earthquakes, mudslides, and other destructive natural disasters may damage the original road network and cause traffic disruption [8], especially plateau mountain area with fewer roads than plain areas, which has fewer alternatives and is more sensitive to natural disasters [9]. While the road network is destroyed or does not exist, decision-makers are required to fully understand the present geographic environment by analyzing the topographical data. Assessing accurately and quickly the prevailing situation and providing reasonable advice for rescue path planning of major disaster emergencies is so crucial that appropriate action can be taken on time [10]. Therefore, planning disaster emergency rescue path based on topographical data is particularly important in a road-free environment.

Nowadays, researchers have carried out a series of exploratory researches on the pan planning problem under the road-free environment. The content of the research mainly includes environmental trafficability analysis and path search algorithm. Environmental trafficability analysis is the first prerequisite for implementing path planning. Liu et al. used a digital elevation model (DEM) to generate various curves to approach the earth's surface through certain rules and to construct mathematical models of 3D maps to describe off-road environments [11]. Yuan et al. used a digital elevation model to simulate complex geomorphologic shapes and established a mathematical model to study the effect of slope on the trafficability and efficiency of vehicle off-road mobility [12]. Wu et al. used the area dominant method to rasterize the surface properties, established the roughness evaluation index of the surface properties, introduced the window movement method to calculate the slope of the terrain in advance, and completed the trafficability analysis by establishing a taboo table [13]. Han et al. established a slope and soil taboo table according to the theoretical analysis and simulation experiments to determine passing characteristics of the terrain-vehicle relationship [14]. However, there are still some shortcomings in the current research: (1) The current research mainly considers the influence of local factors on the traffic situation of relief objects in charge of disaster, and the planned path is difficult to meet the actual needs, so the trafficability analysis should comprehensively consider the combined influence of multiple factors. (2) The traffic capacity of various maneuvering objects differs under the same road-free environment. For example, wheeled vehicles cannot pass through the swamp terrain, while tracked vehicles can pass at low speed. Therefore, the differences in the attribute information of maneuvering objects should be considered when studying the path planning of maneuvering objects, but the available path planning research does not distinguish this, which cannot give full play to the performances of maneuvering objects. In the literature, several search algorithms and methods proposed to conduct scheduled relief processes under various disaster scenarios have been investigated [15–21].

A^∗^ path search algorithm is based on the established environmental model, searching between the starting point and the target point to obtain an optimal path. For the path planning problem under a road-free environment, the main algorithms currently include genetic algorithm, ant colony algorithm, artificial potential energy algorithm, and A^∗^ algorithm.

The A^∗^ algorithm has the advantages of flexibility and a high success rate among them, which is more suitable for global path planning under a road-free environment. Some experts and scholars have currently put forward some improvements to the A^∗^ algorithm. Geo et al. [22] put forward a path planning navigation strategy based on the two-way smoothing A^∗^ algorithm, which improves the real-time of the algorithm by optimizing the cost function of the A^∗^ algorithm and turning its overall search direction to a two-way street. Liu et al. [23, 24] proposed the adaptive controller designs and adaptive fuzzy synchronization of uncertain fractional-order chaotic and nonlinear systems. Jan et al. [25] studied cyber securities by using fuzzy modeling techniques. Xian et al. [26] optimized the A ^∗^ algorithm by introducing three new methods of “guideline,” “key point list,” and “bidirectional search” to solve the storage space occupation problem of the A^∗^ algorithm [26]. Ma et al. used the key point selection strategy for quadratic programming to effectively remove multiple inflection points and redundant nodes, to improve the planning efficiency [27]. However, with the increase of spatial dimension, the computational complexity of the A^∗^ algorithm increases exponentially, and it will eventually face dimension disaster, showing poor scalability, which makes it not perform long-distance emergency rescue path planning tasks in the road-free environment. Based on the characteristics of strong timeliness and the existence of no road network in emergency rescue path planning, this paper carries out research in the context of the shortcomings of path planning in a road-free environment.

Firstly, the traffic analysis of the environment without a road network is carried out. The process includes the use of terrain and geomorphologic data to complete environmental modeling. Secondly, environmental trafficability and traffic efficiency are evaluated by formulating pass evaluation rules after jointly considering the interaction between environment and maneuvering objects and their impact on traffic. Subsequently, the A^∗^ algorithm is improved with the traffic efficiency as the influence weight and timeliness as the goal, which effectively improves the computational efficiency and realizes the rapid path planning in the road-free environment, providing decision-making services for the path planning of disaster emergency rescue. On this basis, the emergency rescue path planning system in the road-free environment is developed and experimentally verified.

The organization of the manuscript is presented as follows: Section 2 deals with the analysis of environmental trafficability based on the grid method to quantify basic terrain and geomorphologic data. A^∗^ algorithm and the improved A^∗^ algorithm have been presented and proposed, respectively, in Section 3. With the A^∗^ algorithm, the heuristic search function is advanced.  Section 4 presents data collection and processing, the phases of data analysis, implementation of the proposed method, and results.  Section 5 concludes the research.

## 2. Analysis of Environmental Trafficability

Environmental trafficability is the basis of path planning under a road-free environment, and it evaluates the trafficability and traffic efficiency of ground maneuvering objects by analyzing the traffic impact of terrain geomorphological data and the performance properties of mobile objects [28]. In this paper, the grid method is used to quantify the basic terrain and geomorphologic data to complete the environmental modeling. Traffic impact is considered based on a relationship between traffic and multiple influencing factors and formulating pass evaluation rules. Afterward, the environmental traffic and traffic efficiency of the maneuvering object are finally calculated.

### 2.1. Modeling of Environment

Environment modeling is the chief prerequisite to realizing path planning under a road-free environment, which means building the corresponding mathematical model through processing and analyzing environmental information for path search and optimization [29]. The visual graph method, grid method, and so on are generally used to construct environmental models [30]. The grid method is widely used in environmental modeling among them since its simple modeling process, which is conducive to calculation and execution, its strong adaptability to the changeable environment, and its ability to represent different obstacles are the advantages concurrently. The main idea of the grid method is to decompose the environmental information discretely and represent it by grid pixels of a certain scale.

**Figure 1:**
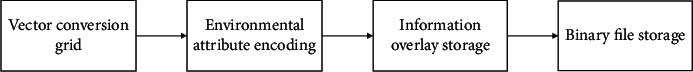
Process of terrain quantification.

To conduct grid modeling, the following steps are followed: converting the vector data of the environment is planned to raster data, then digitally encoding and merging various types of terrain and geomorphologic information corresponding to the grid pixels are conducted, respectively, and finally, the attribute information of the processed raster data is stored as binary files. This storage method greatly improves the efficiency of reading and using raster information, guaranteeing the rapid generation of path planning schemes. The specific process of environmental modeling is shown in Figure 1.

#### 2.1.1. Conversion Grid Vector Information

Vector conversion grid refers to the conversion of vector geometry information to grid pixels, providing data support for the implementation of the path planning algorithm in the road-free environment, realized by calling open-source Geospatial Data Abstraction Library (GDAL).

#### 2.1.2. Encoding of Environmental Attribute

Environmental attribute encoding refers to encoding the environmental attribute information corresponding to raster data to the corresponding integer number. Types of attribute field data of grid data are divided into floating-point and string. Floating-point fields can be directly assigned to pixels after conversion, such as water depth, vegetation spacing, and slope data, while fields of string type, such as vegetation, geology, and disasters, need to be encoded as appointed integer numbers and then assigned to pixels, for example, the tree in the vegetation type, encoded as a number 1 by the attribute. When data is read, the pixel value is 1, so the vegetation type can be extracted as the tree.

#### 2.1.3. Storage of Information Overlay

Information overlay storage refers to combining and storing the integer data encoded by environmental attributes for representing various types of influencing factors of traffic conditions, for example, data 10000000, single-bit stored disaster data, ten-bit stored geological data, and one hundred-bit stored vegetation type data. For slope data, it occupies three digits and retains one decimal place. For example, 20.5 degrees is stored as 205, of which 005 represents 0.5 degrees. These three digits occupy thousands, ten thousand, and one hundred thousand of integer data. By analogy, all traffic condition factor data are merged into long integer data and stored in a binary file.

### 2.2. The Rules of Passing Evaluation

The passing evaluation rule implies that the path planning scheme can be used and practical and thus plays a very important role in path planning under the road-free environment. This paper comprehensively considers the pass-through relationship between the environment model and maneuvering object model and the environment model and kinematic constraint and thus puts forward the pass evaluation index system, which has an impact on environmental accessibility. Its influencing factors are presented in Table 1. While the environmental model refers to the perception of the environment, including the surface cover and the geometry of the surface, the maneuvering object model refers to its basic physical properties, and the maneuvering kinematic constraint of an object describes its permissible state of motion.

Evaluation factors in the evaluation index system are not independent. Some factors interact with each other. For example, the width of the car body determines the minimum spacing that can pass through the trees, and the chassis height affects its wading capacity so that it determines the maximum average depth of water that can pass through and the climbing ability determines the maximum slope that can pass through the terrain. In addition, geologic and disaster types can also affect the speed of vehicles.

In this paper, the pass evaluation rules have been formulated with combinations of the effects mentioned above, which are presented in Table 2. In the table, the influencing weight *W* is the weight of all the influencing factors, and the greater the weight is, the greater the impact it has on the passing efficiency of the ground maneuvering object. The velocity coefficient *fv* is used to measure the extent of each influencing factor's affection to the travel speed of the maneuvering object, which can be adjusted flexibly according to the actual situation, with a value in the range of [0-1]. The velocity coefficient of 1 indicates that the maneuvering object can pass at the maximum velocity, and a velocity coefficient of 0 means that it is impassable.

In the rules of general assessment setup in this paper, the main factors affecting the passing efficiency of maneuvering objects are vegetation, water system, geology, disaster, and the DEM. Based on the principle of analytic hierarchy process, the influence degree of each factor on the traffic efficiency is obtained by utilizing data, expert scoring, and investigation in the form of the judgmental matrix employing a pairwise relationship between the five factors, which is presented in (1) [31–33].

(1) $$\begin{array}{c} A=\left[\begin{array}{ccccc} 1 & 1/2 & 1/4 & 1/2 & 1/3\\ 2 & 1 & 1/3 & 1/3 & 1/2\\ 4 & 3 & 1 & 1/2 & 2\\ 2 & 3 & 2 & 1 & 3\\ 3 & 2 & 1/2 & 1/3 & 1 \end{array}\right], \end{array}$$

(2) $$\begin{array}{c} W=\left[0.087，0.110，0.275，0.357，0.171\right], \end{array}$$

(3) $$\begin{array}{c} \lambda_{\max}=\sum_{1}^{n}\frac{{\left[AW\right]}_{i}}{nw_{i}}, \end{array}$$

(4) $$\begin{array}{c} \text{CI}=\frac{\left(\lambda_{\max}-n\right)}{\left(n-1\right)}, \end{array}$$

(5) $$\begin{array}{c} \text{CR}=\frac{\text{CI}}{\text{RI}}. \end{array}$$

After calculation, the corresponding eigenvectors of matrix A are obtained, as shown in formula (2), and then by formulas (3) and (4), *λ*_max_=5.292 and *CI*=0.073 are obtained and *n* in the formula refers to the number of influencing factors. The *RI* value is the consistency test value of the matrix, and the value of the fifth-order matrix is 1.12. After substituting it into equation (5), *CR*=0.065 < 0.10 is obtained, indicating that the judgmental matrix A satisfies the consistency test, so the influence weights of vegetation, water system, geology, disaster, and the DEM have been found to be 0.087, 0.110, 0.275, 0.357, and 0.171, respectively.

The wheeled transport vehicle as an emergency rescue material is utilized for the actual situation. This paper evaluates the velocity coefficient *fv* of the maneuvering object employing the impact of factors such as the collection of data, scores obtained by experts, and analysis of data [31-33]. Table 2 presents all values.

### 2.3. Trafficability and Passing Efficiency

Trafficability and passing efficiency are the results of environmental trafficability analysis, which can be calculated using the established passing evaluation rules after environmental modeling and can be used for path searching in a road-free environment.

#### 2.3.1. Trafficability

**Figure 2:**
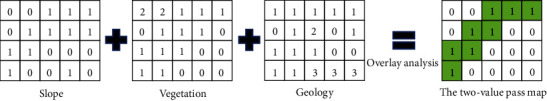
Calculation of passing map.

In the passing domain model, the passable value is used to represent the trafficability of the maneuvering object, the 0 value is inaccessible, the non-0 value is accessible, and the values between 0 and 1 are used to distinguish different maneuvering objects, for example, vegetation. The values 1 and 2 of the vegetation part in Figure 2 represent different vegetation types, respectively. Finally, the stack analysis based on the array of traffic domains of each influencing factor is carried out to obtain the binary traffic map for path planning, and the traffic value is either 0 or 1. When the traffic conditions corresponding to each influencing factor are accessible, the traffic value is equal to 1, indicating that the environmental traffic conditions are assessed as good; when the traffic condition corresponding to the influence factor is impassable, the pixel is an impassable area, and the traffic value takes 0.

#### 2.3.2. Passing Efficiency

Passing efficiency can be measured based on the time used by the maneuvering object through the raster pixel. The calculation of the passing time needs to take into account the influence of environmental factors on the speed of the maneuvering object's movement and establish the formula for calculating the passing speed. According to the size of the pixel and the speed of the moving pixel, the calculation formula of the transit time of adjacent pixels is constructed to support the path transit time calculation.

The maximum travel speed of the maneuvering object is *V*_Max_, the velocity coefficient is denoted by *fv* (see Table 2), and the passing speed of a single influencing factor denoted by *Vx* is defined by

(6) $$\begin{array}{c} \text{Vx}=V_{\text{Max}}\times fv. \end{array}$$

*W*_vegetation_, *W*_water_, *W*_geology_, *W*_DEM_, and *W*_disaster _ represent the weight of vegetation, water system, geology, the DEM, and disaster factors in all the factors affected, respectively. When equation (7) is written, we abbreviate the words with the first letter of each word, namely, *V*, *W*, *G*, DEM, and *D*, respectively. The maneuvering object in each cell passing velocity coefficient *fv*_*t*_ can be defined by (based on the actual cell influence factors)

(7) $$\begin{array}{c} f_{v_{t}}=\frac{W_{V}\times f_{v_{V}}+W_{W}\times f_{v_{W}}+W_{G}\times f_{v_{G}}+W_{\text{DEM}}\times f_{v_{\text{DEM}}}+W_{D}\times f_{v_{D}}}{W_{V}+W_{W}+W_{G}+W_{\text{DEM}}+W_{D}}. \end{array}$$

By substituting equations (5) into (6), the velocity V of the maneuvering object in each pixel can be expressed by

(8) $$\begin{array}{c} V=V_{\text{Max}}\times fv_{t}. \end{array}$$

**Figure 3:**
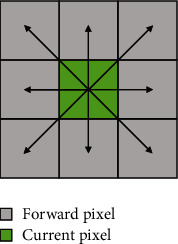
Eight-direction graph of four-corner grid.

Figure 3 depicts the maneuvering object that can move in eight directions in the current cell. Let *R* be the pixel length and width, *V* be the current pixel velocity, and *V*_1_ be the forward pixel velocity. The passing time for adjacent cells is calculated as follows:

(9) $$\begin{array}{c} 1\text{.Time of straight distance: T}=\frac{\left(R/2\right)}{V}+\frac{\left(R/2\right)}{V_{1}}\\ =\frac{R\left(V+V_{1}\right)}{2VV_{1}}. \end{array}$$

(10) $$\begin{array}{c} 2\text{.Time of oblique distance }{\text{:T}}^{'}=\frac{\left(\sqrt{2}R/2\right)}{V}+\frac{\left(\sqrt{2}R/2\right)}{V_{1}}\\ =\frac{\sqrt{2}R\left(V+V_{1}\right)}{2VV_{1}}. \end{array}$$

## 3. The Improved A^∗^ algorithm

### 3.1. The Conventional A^∗^ Algorithm

A ^∗^ algorithm that uses a heuristic function for path search is suitable for path planning with known global environment information. A ^∗^ algorithm uses cost function for path planning whose cost function is defined by

(11) $$\begin{array}{c} F\left(n\right)=G\left(n\right)+H\left(n\right). \end{array}$$

In equation (11), *F*(*n*) represents the cost assessment from the starting grid point to the target point via any node *n*, *G*(*n*) is the actual distance assessment from the starting point *s* to the grid *n*, and *H*(*n*) represents the European distance assessment from the grid *n* to the endpoint e. In equation (12), *H*(*n*) is defined as follows:

(12) $$\begin{array}{c} H\left(n\right)=\text{dist}\left(n,e\right)\\ =\sqrt{\left(e_{x}-n_{x}\right)+\left(e_{y}-n_{y}\right)}, \end{array}$$

where *e*_*x*_ and *e*_*y*_ in the formula are the *x* coordinates and *y* coordinates of the endpoint e and the *n*_*x*_ and *n*_*y*_ are the *x* coordinates and *y* coordinates of the current point *n*.

**Figure 4:**
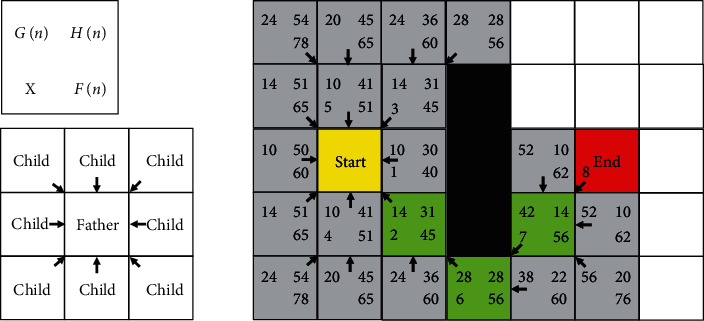
The simplified model for the search of the evaluation function.

The simplified version of the conventional A ^∗^ search algorithm is shown in Figure 4. The initial cell is used as a search cell, then searching and calculating the *F*(*n*) value of the adjacent child node is conducted, and all the *F*(*n*) values of the searched nodes in the list are recorded.

The list is arranged by the *F*(*n*) value and determines the priority of the nodes. The smaller the *F*(*n*) value is, the higher the priority is searched. Repeating this process is necessary until the path planning is completed, so the final path is generated.

In Figure 4, the upper-left corner number of each grid pixel represents *G*(*n*), the upper-right corner number represents *H*(*n*), the lower-right corner number represents *F*(*n*), the lower-left corner number *X* represents the *X* search pixel selected according to *F*(*n*) value. The arrow designates a movement from the subpixel to the parent pixel.

### 3.2. The Improved A^∗^ Algorithm

**Figure 5:**
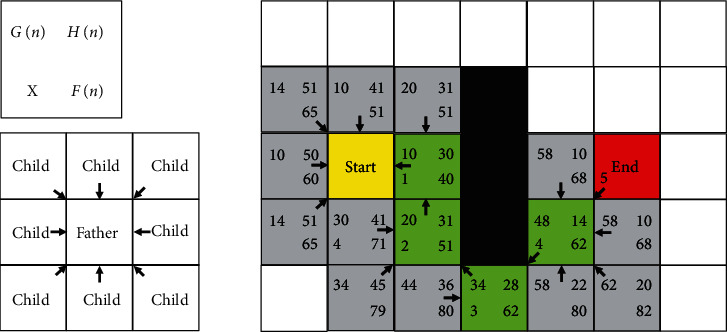
The simplified model by the improved search algorithm.

Based on the comprehensive consideration of the efficiency of maneuvering object passage and the efficiency of algorithm searching, an improved A^∗^ algorithm is proposed. The main improvements can be summarized as follows:

(1) The traffic cost of different maneuvering objects is affected by the surface type and the traffic capacity of the maneuvering object itself; this paper improves the original heuristic function, and the formula is as follows:
  (13) $$\begin{array}{c} F_{sh}\left(n\right)=G_{q}\left(n\right)+H_{t}\left(n\right). \end{array}$$
*G*_*q*_(*n*) denotes the sum of the costs from the starting point *s* of the maneuvering object to the grid *n*, and the generation value of each pixel is obtained by the passing velocity coefficient *fv*_*t*_ in equation (7). The formula is defined by
  (14) $$\begin{array}{c} G_{q}\left(n\right)=\sum_{s}^{n}\left(1-fv_{t}\right). \end{array}$$
(2) The improved A ^∗^ algorithm takes into account the elevation difference, and the relatively flat terrain is preferred to move forward. *H*_*t*_(*n*) is obtained by the Euclidean distance and elevation difference, and the calculation is shown in equation (15), where *H*(*n*) is the Euclidean distance from the current point *n* to the endpoint *e* , which plays a pointing role. The calculation is shown in equation (12). *H*_*d*_ is the absolute value of the difference between the parent node elevation *H*_*f*_ and the current node elevation *H*_*C*_, which is used to evaluate the terrain fluctuation, and the calculation is shown in equation (16).
  (15) $$\begin{array}{c} H_{t}\left(n\right)=H_{d}+H\left(n\right), \end{array}$$
  (16) $$\begin{array}{c} H_{d}=\left|H_{f}-H_{C}\right|. \end{array}$$
(3) Improving the A^∗^ search algorithm leads the model to be simplified as shown in Figure 5. The initial cell is used as a search cell, and the *F*(*n*) value of the adjacent child node is searched and calculated. Then, the cell with the smallest *F*(*n*) value is selected as the current search cell. Repeating this process is needed until the path planning is completed and the final path is generated.

In Figure 5, the left upper corner number of each grid pixel represents *G*_*q*_(*n*), the right upper corner number represents *H*_*t*_(*n*), the right lower corner number represents *F*_*sh*_(*n*), the left lower corner number *X* represents the *X* search pixel, and the arrow points from the subpixel to the parent pixel are found.

In this simplified model, *G*_*q*_(*n*) and *H*_*t*_(*n*) are represented by distance. When compared with the simplified model of the conventional A ^∗^ search algorithm, the improved A ^∗^ algorithm is seen to be not optimal in the planning path distance effect, but the computational efficiency is improved. Particularly, with the increase of the data scale, the advantage of its computational efficiency will be more obvious.

The detailed steps of the improved A ^∗^ algorithm are presented in Table 3.

## 4. Experiments and Analysis

To verify the validity of the trafficability analysis and algorithm, the rescue path planning system of an environmental emergency for a road-free environmental emergency is developed, and experimental analysis is carried out.

### 4.1. Data Preparation

#### 4.1.1. Geographical Environment Data

**Figure 6:**
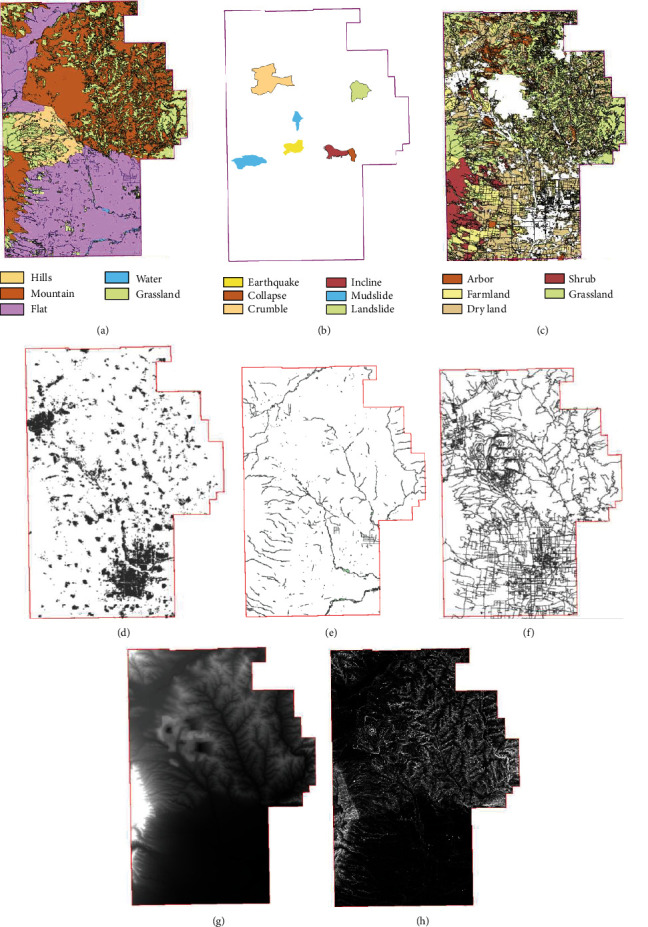
No road network environmental data. (a) Geology. (b) Disaster. (c) Vegetation. (d) Residential area. (e) Water system. (f) Road. (g) DEM. (h) Slope.

This experiment constructs the nonroad network environment through the topography data of Shuozhou City, Shanxi Province. Shuozhou City is fully characterized as a loess-covered mountain terrain plateau with complex and diverse natural conditions and obvious transitional nature, which is suitable for simulating complex nonroad network environments. The data used include the raster DEM data and vector data of vegetation, water system, geology, disaster, and residential area depicted in Figure 6.

#### 4.1.2. Data of Maneuvering Object

The maneuvering object is called a wheeled emergency relief vehicle, and the performance data of the maneuvering object are presented in Table 4.

#### 4.1.3. Results and Analysis

The environment is modeled using the provided geographic environment data. The road-free environment model has a grid resolution of 2 meters and about 200 million grid cells. Subsequently, the experimental data are processed by the established pass evaluation rules to generate the passing map of wheeled emergency rescue vehicles without a road network environment, and then the starting point and target point of emergency rescue path planning are set. The results of the experiment are analyzed.

### 4.2. Trafficability Results and Analysis

**Figure 7:**
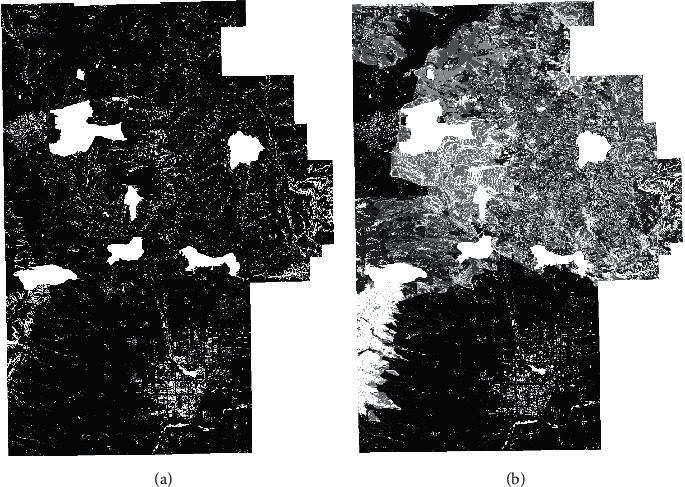
The results of pervasiveness analysis. (a) The two-value passing map. (b) Passing efficiency map.

Figure 7 depicts the result of the trafficability analysis of the wheeled emergency relief vehicle.  Figure 7(a) denotes the binary pass diagram of the maneuvering object, where the black area is the accessible area and the white area is the inaccessible area. When compared with Figures 6(b), 6(d), 6(e), and 6(h), the areas of the residents' land, water system, geological disaster area, and slope value not meeting the prevailing conditions which are determined as impassable by the rules of passing evaluation can be seen.  Figure 7(b) depicts the passing efficiency map, which reflects the passing efficiency of the maneuvering object through the color depth of the passable area. The closer to black the color is, the higher the passing efficiency of the maneuvering object is. On the contrary, the closer to white the color is, the lower the maneuvering object's passing efficiency is. When compared to Figure 6, the mountain and hilly areas are more difficult to pass, so the color is close to white, while the more accessible in flatlands are close to black.

**Figure 8:**
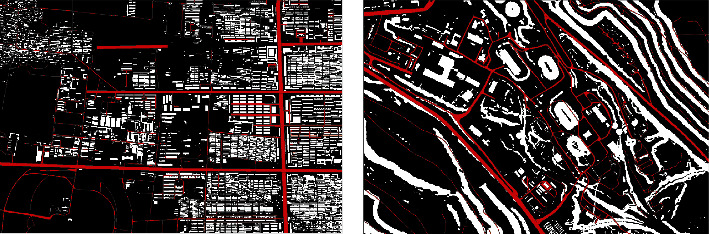
Comparison of existing roads.

Covering existing road data on the road map is depicted in Figure 6. The red area is the road data of the area. Besides, Figure 8 depicts most of the existing roads that are in the passable area, which suggests that the maneuvering object passage map generated by this method has reliability.

### 4.3. The Results of the Path Planning Analysis

**Figure 9:**
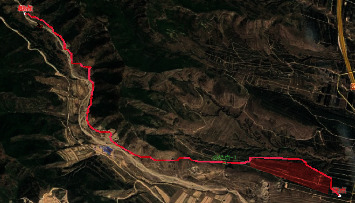
The effect of two-dimensional perspective planning.

**Figure 10:**
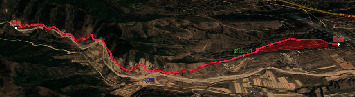
3D perspective planning effect.

While Figure 9 shows a two-dimensional effect of the perspective planning, Figure 10 depicts the three-dimensional effect of it. Through visual analysis, the planning path avoids impassable areas and follows the valley.

The improved A^∗^ algorithm is compared with the conventional A^∗^ algorithm, and the comparison data are shown in Table 5. The search time of the conventional A^∗^ algorithm increases exponentially and eventually faces the dimensional disaster; the phenomenon of computational stagnation or collapse occurs when the length of the search path grows. By comparing the search time data, the search efficiency of the improved A^∗^ algorithm is greatly improved, especially in long-distance path search. When comparing the path distance data, the path distance planned by the improved A^∗^ algorithm that is relatively longer is found, and the phenomenon is analyzed based on the algorithm design level. The improved A^∗^ algorithm with strong timeliness that takes into account the influences of both slope and the weight of passing efficiency as a goal to achieve the task will bypass the planning scheme when a high slope difficult to pass is encountered in the terrain. This can be supported by the data in Table 5. When comparing the total time data, it is found that the improved A^∗^ algorithm embodies the characteristics of strong timeliness. Besides, by comparing some path distances and estimated time data results, it is discovered that the estimated traffic time of A^∗^ is shorter, although the path distance is longer than the traditional A^∗^ algorithm. When most of the slope proportion and geological type proportion data are compared, it is found that the improved A^∗^ algorithm tends to choose a smoother and easy-to-pass terrain. In summary, the improved A^∗^ algorithm has a great theoretical significance and application value to the rapid planning of rescue paths for disaster emergencies in a road-free environment.

## 5. Conclusion

The rapid planning of the path of disaster relief personnel, vehicles, and other mobile objects in the road-free environment is of great significance to ensure the safety of the lives of the affected people and the supplies of materials. Therefore, since planning those efforts in the current road-free environment looks more complicated than the areas having road network, optimizing the characteristics of emergency rescue path planning with maneuvering objects requires putting forward a better implementable method for disaster emergency rescue path planning in the road-free environment, which mainly contains the environmental trafficability analysis dealing with the issues of timeliness and traffic efficiency. Thus, the improvement of the currently implemented A^∗^ algorithm is proposed in this paper, which is called the improved A^∗^ algorithm. The environmental trafficability analysis of the road-free network has laid a data foundation to enhance the A^∗^ algorithm.

The improvement is constructed based on the issues that have not been visited yet, which are called the trafficability analysis and strong timeliness. To resolve those issues, the rapid planning of the emergency rescue paths that mobile objects follow in the road-free environment is conducted based on the trafficability of the area. Thus, the traffic efficiency and timeliness are improved by heuristic search optimization and grid method by focusing on the influential weights. On the other hand, the A^∗^ algorithm generally takes a longer time to find a rescue path than does the improved A^∗^ algorithm when long distance is a concern. Besides, the A^∗^ algorithm generally fails under the search for long distance or does not generate practical solutions.

By experimenting and analyzing the method using the data of Shuozhou City, Shanxi Province, the results show that the environmental trafficability data of the road-free network have certain rationality and reliability, while the search efficiency of the improved A^∗^ algorithm is greatly improved, the time-sensitive characteristics of the character and emergency rescue are greatly improved, and the influence of slope and weights of passing efficiency is taken into account by the improved A^∗^ algorithm, which makes the planned path more flexible and practical.

The research has contributed a certain theoretical significance and application value, which can provide a reference for the rapid planning of disaster emergency rescue paths in the environment of no road network.

Although a set of preliminary feasible solutions have been formed in the planning and research work of disaster emergency rescue paths in the environment of no road network, further research is needed to enhance the proposed algorithm. The next round of research will be focusing on improving the rules of passing an evaluation, optimizing the algorithm of the path planning, and advancing the rapid planning ability of disaster emergency rescue paths in road-free environments.

## Figures and Tables

**Table 1:** Evaluation index that influences factors of traffic condition.

Influencer	Evaluation indicator	Evaluation factor
Environmental factors	Surface cover	Vegetation
		Water system
		Geology
		Disaster
	Surface geometry	Slope
		Elevation

Maneuvering object factors	Maneuvering object model	Body width
		Chassis height
	Motion constraints of maneuvering objects	Climbing ability
		Wading capacity

**Table 2:** Rules for general evaluation.

Influencer	Impact weight	Index	Factor		Velocity coefficient *fv*
Vegetation (body width)	0.087	Type	Grassland		0.70
			Dryland		0.80
			Farmland		0.50
		The average spacing of shrubs	<motorized object body width		0
			≥motorized object body width	<3 m	0
				≥3 m	0.20
		The average spacing of trees	<motorized object body width		0
			≥motorized object body width	<3 m	0
				≥3 m	0.20

Water system (chassis height)	0.110	Average depth	<the wading capability of the motorized object	<30 cm	0.30
				≥30 cm	0.10
			≥the wading capability of the mobile object		0
		Bottom	Mud		0
			Sand		0.20
			Gravel		0.30

Geology	0.275	Type	Flat ground		1.00
			Hill		0.20
			Mountain		0.10
			Gobi		0.25
			Beach		0
			Swamp		0
			Desert		0.40

Disaster	0.357	Type	Mudslide		0
			Landslide		0
			Slope		0.10
			Crumble		0
			Collapse		0
			Earthquake		0.20

DEM (climbing ability)	0.171	Slope	<the climbing ability of the motorized object	<10°	0.80
				10°–30°	0.30
				>30°	0
			≥the climbing ability of the motorized object		0
		Elevation	<2000 m		0.89
			2000 m–5000 m		0.65
			>5000 m		0.26

**Table 3:** The calculation steps of the improved A^∗^ algorithm.

The process for the improved A^∗^ algorithm
Input: Environment digital model, starting point, and target point
(1) Initialize the cell
(2) If initialization is marked as 0, indicate that the grid has not been evaluated
(3) Else initialization is marked as 1, indicating that it is represented as a point on the path or impassable
(4) Create a container m_openList that holds the path node
(5) Add the starting point to the m_openList as the current node
(6) While (the current node is not the target point)
(7) View the current node adjacent to passable child nodes
(8) If (there are child nodes in m_openList)
(9) There is a cross in the path, so remove the cross section
(10) Else if (child nodes exist passable)
(11) Calculate the *F*_*sh*_(*n*) value of the adjacent passable subpoint; see formula (13)
(12) Select the adjacent passable subpoint with the smallest *F*_*sh*_(*n*) value
(13) Add to the m_openList and use it as the current point of the path
(14) Else
(15) Return the previous node and select the minimum *F*_*sh*_(*n*) value among the remaining child nodes
(16) Falling back to the starting point means that no path has been found
(17) Return failed to find a way
(18) Return m_openList
(19) Traverse the nodes on the path to get path information
(20) Calculate the estimated passing time of the path by using formulas (9) and (10)
Output: The path from the starting point to the target point

**Table 4:** Performance data of maneuverable objects.

Object type	Maximum mileage (km)	Climbing ability (°)	Wading depth (cm)	Maximum speed (kph)	Size	Chassis height (m)	Suitable geology
Wheeled emergency relief vehicle	200	30	30	90	4.2 m∗1.8 m	1.3	See Table 2 for details

**Table 5:** The comparison data of the algorithm.

	The conventional A^∗^ algorithm								The improved A^∗^ algorithm							
	Path distance (km)	Time loss (h-m-s)			Slope proportion (%)		The proportion of geological types (%)		Path length (km)	Time loss (m-s)			Slope proportion (%)		The proportion of geological types (%)	
		Search time	Preestimate time	Total time	0°–20°	20°–30°				Search time	Preestimate time	Total time	0°–20°	20°–30°		
Short distance	0.757	1 m 47 s	46 s	2 m 33 s	100	0	Grass 99	Chu 1	0.782	2 s	48 s	50 s	99	1	Grass 100	
	0.942	51 s	1 m 31 s	2 m 22 s	76	24	Grass 1	Mountain 99	1.048	5 s	1 m 26 s	1 m 31 s	100	0	Grass 16	Mountain 84
	0.965	47 s	1 m 17 s	2 m 4 s	83	17	Grass 11	Mountain 89	1.075	2 s	1 m 8 s	1 m 10 s	100	0	Grass 52	Mountain 48

Shorter distance	1.866	2 m	2 m 43 s	4 m 43 s	81	19	Grass 6	Mountain 94	2.01	6 s	2 m 48 s	2 m 54 s	83	17	Grass 25	Mountain 75
	1.876	27 m 36 s	2 m 11 s	29 m 47 s	67	33	Grass 65	Mountain 35	2.235	1 s	2 m 23 s	2 m 24 s	70	30	Grass 79	Mountain 21
	2.032	17 m 49 s	2 m 29 s	20 m 18 s	80	20	Grass 43	Mountain 57	2.416	4 s	2 m 48 s	2 m 52 s	76	24	Grass 66	Mountain 34

Medium distance	3.18	103 m 26 s	3 m 35 s	107 m 1 s	90	10	Grass 63	Chu 37	3.2	5 s	3 m 55 s	4 m 0 s	99	1	Grass 52	Chu 48
	4.887	201 m 11 s	5 m 46 s	206 m 57 s	70	30	Grass 56	Mountain 44	5.594	5 s	6 m 19 s	6 m 24 s	66	34	Grass 68	Mountain 32
	4.502	292 m 13 s	5 m 38 s	297 m 51 s	84	16	Grass 31	Mountain 69	5.65	23 s	6 m 17 s	6 m 40 s	81	19	Grass 77	Mountain 23

Long distance	—	>5 h	>5 h	>5 h	—	—	—	—	11.069	7 s	13 m 22 s	13 m 29 s	30	70	Grass 54	Mountain 46
	—	>5 h	>5 h	>5 h	—	—	—	—	19.202	10 s	22 m 38 s	22 m 48 s	76	24	Grass 55	Mountain 45
	—	>5 h	>5 h	>5 h	—	—	—	—	41.121	2 m 16 s	44 m 42 s	46 m 58 s	83	17	Grass 60	Mountain 40

## Data Availability

The data used to support and prove the findings of this study are available from the corresponding author upon request.

## References

[1] Du Z., Li Y., Zhang Y., Tan Y., Zhao W. (2020). Knowledge graph construction method on natural disaster emergency. *Journal of Wuhan University (Natural Science Edition)*.

[2] Tan X., Lin Y. (2014). Study of disaster relief path planning based on improved genetic ant colony hybrid algorithm. *Computer Engineering and Design*.

[3] Wang T., Guo Q., Zhang Y. (2018). Route optimization problem on goods and materials distribution in post-earthquake relief. *Computer simulation*.

[4] Yuan Y., Wang D. (2008). Path selection model and algorithm for emergency logistics management. *Computers & Industrial Engineering*.

[5] Jotshi A., Gong Q., Battar R. (2008). Dispatching and routing of emergency vehicles in disaster mitigation using data fusion. *Socio-Economic Planning Sciences*.

[6] Vodák R., Bil M., Krivánková Z. (2018). A modified ant colony optimization algorithm to increase the speed of the road network recovery process after disasters. *International Journal of Disaster Risk Reduction*.

[7] Zhu J., Liu S., Ghosh S. (2019). Model and algorithm of routes planning for emergency relief distribution in disaster management with disaster information update. *Journal of Combinatorial Optimization*.

[8] Su D., Guo Z. (2021). Selection model of emergency rescue passageway after highway disasters. *Highways*.

[9] Li J., Liu J., Guo L., Wang W. (2020). Research on emergency rescue plan of plateau area after an earthquake based on road accessibility. *Journal of Natural Disasters*.

[10] Yan F., Tang X., Guo L., Shi M., Yu L., Wei H. (2021). Geological environment background of Laoling area of Yalu River and the trafficability analysis of emergency relief materials for major disasters. *Safety and Environmental Engineering*.

[11] Liu Q., Zhao L., Tan Z., Chen W. (2017). Global path planning for autonomous vehicles in off-road environment via an A-star algorithm. *International Journal of Vehicle Autonomous Systems*.

[12] Yuan R., Chan G., Wang B. (2017). Analysis of influence of terrain slope on off-road maneuvering time. *Geographic Information World*.

[13] Wu T., Xu J., Liu J., Liao W. (2013). Research of cross-country path planning based on improved A^∗^ algorithm. *Computer application research*.

[14] Han W., Zhang H., Wang K., Zhang C. (2018). Off-road path planning based on improved ant colony. *Algorithms*.

[15] Ding Z., Zhao Z., Liu D., Cao Y. (2021). Multi-objective scheduling of reliefs logistics base on swarm intelligence algorithms and Spatio-temporal traffic flow. *Journal of Safety Science and Resilience*.

[16] Xu N., Zhang Q., Zhang H., Hong M., Akerkard R., Lianga Y. (2019). Global optimization for multi-stage construction of rescue units in disaster response. *Sustainable Cities and Society*.

[17] Xu X., Zhang L., Trovati M. (2021). PERMS: an efficient rescue route planning system in disasters. *Applied Soft Computing*.

[18] Yu X., Li C., Zhou J. F. (2020). A constrained differential evolution algorithm to solve UAV path planning in disaster scenarios. *Knowledge-Based Systems*.

[19] Shi X., Gai W., Xu K. (2022). Bi-objective rescue path selection optimization for mine fires based on quantitative risk assessment. *Safety Science*.

[20] Yu X., Li C., Gary Yen G. G. (2021). A knee-guided differential evolution algorithm for unmanned aerial vehicle path planning in disaster management. *Applied Soft Computing*.

[21] Chou J. S., Cheng M. Y., Hsieh Y. M., Yang I. T., Hsu H. T. (2019). Optimal path planning in real time for dynamic building fire rescue operations using wireless sensors and visual guidance. *Automation in Construction*.

[22] Geo Y. F., Zhang M., Shen C., Guan X. (2021). Bi-directional smooth A-star algorithm for navigation planning of mobile robots. *Scientia Sinica Technologica*.

[23] Liu H., Li S., Li G., Wang H. (2018). Adaptive controller design for a class of uncertain fractional-order nonlinear systems: an adaptive fuzzy approach. *International Journal of Fuzzy Systems*.

[24] Liu H., Chen Y., Li G., Xiang W., Xu G. (2017). Adaptive fuzzy synchronization of fractional-order chaotic (hyperchaotic) systems with input saturation and unknown parameters. *Complexity*.

[25] Jan N., Nasir A., Alhilal M., Khan S., Pamucar D., Alothaim A. (2021). Investigation of cyber-security and cyber-crimes in oil and gas sectors using the innovative structures of complex intuitionistic fuzzy relations. *Entropy*.

[26] Xian T. G., Yukun Z., Xinxin J. (2021). Improved A-star algorithm for robot path planning in a static environment. *Journal of Physics Conference Series*.

[27] Ma Z., Zhao J., Zhang Y., Li L. L., Lin H. C. Development of path planning approach based on improved A-star algorithm in AGV system.

[28] Papadakis P. (2013). Terrain traversability analysis methods for unmanned ground vehicles: a survey, Engineering Applications of Artificial Intelligence. *Engineering Applications of Artificial Intelligence*.

[29] Janet J. A., Luo R. C., Kay M. G. The essential visibility graph: an approach to global motion planning for autonomous mobile robots.

[30] Guo Y., Meng Q., Kong F., Lyu W. (2020). Research status and prospects of AUV path planning algorithm. *Computer Science and Exploration*.

[31] Fan L. *Research on Cross-Country Path Planning Technology Based on a Hexagonal Grid*.

[32] Hua Z. W., Jian T. G., Lan W. Y. (2013). Joint Operations Technology Infrastructure Textbook Series: Introduction to the Battlefield Environment.

[33] Gregory E. H. (1918). Military Geology and Topography, A Presentation of Certain Phases of Geology, Geography and Topography for Military Purposes.

